# Use of non-selective β-blockers is associated with decreased tumor proliferative indices in early stage breast cancer

**DOI:** 10.18632/oncotarget.14119

**Published:** 2016-12-23

**Authors:** Alexa Montoya, Clarissa N. Amaya, Andres Belmont, Nabih Diab, Richard Trevino, Geri Villanueva, Steven Rains, Luis A. Sanchez, Nabeel Badri, Salman Otoukesh, Ali Khammanivong, Danielle Liss, Sarah T. Baca, Renato J. Aguilera, Erin B. Dickerson, Alireza Torabi, Alok K. Dwivedi, Aamer Abbas, Karinn Chambers, Brad A. Bryan, Zeina Nahleh

**Affiliations:** ^1^ Department of Biomedical Sciences, Texas Tech University Health Sciences Center, El Paso, Texas, USA; ^2^ Department of Biology, University of Texas, El Paso, Texas, USA; ^3^ Paul L. Foster School of Medicine, Texas Tech University Health Sciences Center, El Paso, Texas, USA; ^4^ Department of Hematology/Oncology, Loma Linda University Health Sciences Center, Loma Linda, California, USA; ^5^ Department of Veterinary Clinical Sciences, University of Minnesota, Saint Paul, Minnesota, USA; ^6^ Border Biomedical Research Center, University of Texas, El Paso, Texas, USA; ^7^ Masonic Cancer Center, University of Minnesota, Minneapolis, Minnesota, USA; ^8^ Department of Pathology, Texas Tech University Health Sciences Center, El Paso, Texas, USA; ^9^ Division of Biostatistics and Epidemiology, Texas Tech University Health Sciences Center, El Paso, Texas, USA; ^10^ Department of Surgery, Texas Tech University Health Sciences Center, El Paso, Texas, USA

**Keywords:** beta blocker, propranolol, breast cancer, proliferation, Ki-67

## Abstract

Previous studies suggest beta-adrenergic receptor (β-AR) antagonists (β-blockers) decrease breast cancer progression, tumor metastasis, and patient mortality; however the mechanism for this is unknown. Immunohistochemical analysis of normal and malignant breast tissue revealed overexpression of β1-AR and β3-AR in breast cancer. A retrospective cross-sectional study of 404 breast cancer patients was performed to determine the effect of β-blocker usage on tumor proliferation. Our analysis revealed that non-selective β-blockers, but not selective β-blockers, reduced tumor proliferation by 66% (*p* < 0.0001) in early stage breast cancer compared to non-users. We tested the efficacy of propranolol on an early stage breast cancer patient, and quantified the tumor proliferative index before and after treatment, revealing a propranolol-mediated 23% reduction (*p* = 0.02) in Ki67 positive tumor cells over a three-week period. The anti-proliferative effects of β-blockers were measured in a panel of breast cancer lines, demonstrating that mammary epithelial cells were resistant to propranolol, and that most breast cancer cell lines displayed dose dependent viability decreases following treatment. Selective β-blockers alone or in combination were not as effective as propranolol at reducing breast cancer cell proliferation. Molecular analysis revealed that propranolol treatment of the SK-BR-3 breast cancer line, which showed high sensitivity to beta blockade, led to a reduction in Ki67 protein expression, decreased phosphorylation of the mitogenic signaling regulators p44/42 MAPK, p38 MAPK, JNK, and CREB, increased phosphorylation of the cell survival/apoptosis regulators AKT, p53, and GSK3β. In conclusion, use of non-selective β-blockers in patients with early stage breast cancer may lead to decreased tumor proliferation.

## INTRODUCTION

Despite great advances in treatment options over the past two decades, breast cancer is still a deadly disease claiming many lives. Approximately 1.7 million women worldwide are diagnosed with breast cancer each year and one third of these women will die of this disease [1] (http://globocan.iarc.fr/Pages/fact_sheets_cancer.aspx). Breast cancer now represents a quarter of all cancers in women worldwide and is a leading cause of cancer death in less developed countries [2]. This is partly because clinical advances, especially expensive new drugs used to combat the disease, are not reaching women living in these regions [3]. Therefore, identifying more affordable treatment options for disadvantaged patients with breast cancer remains highly desirable.

The catecholamines, epinephrine and norepinephrine, stimulate cell membrane-spanning β-ARs and mediate a variety of physiological responses including vascular smooth muscle relaxation, bronchodilation, and other processes responsive to the sympathetic nervous system [4]. Due to their roles in modulating these processes, β-AR antagonists (β-blockers) have been used for decades to manage cardiac arrhythmias, protect against myocardial infarction, and treat hypertension [5]. Recently, several retrospective analyses of data from patients diagnosed with carcinomas have shown note-worthy responses to β-blockade including reduced cancer risk, reduced metastasis, reduced tumor-associated patient mortality, and increased progression free survival [6–15]. In contrast, other studies have found no evidence to suggest that β-blockers prevent cancer occurrence or risk of death [16–18]. Our labs have spearheaded efforts to examine the mechanism of action and clinical use of β-blockers against tumors and have reported that β-blockade disrupts proliferation, survival, migration, cell-to-matrix attachment, and global transcriptional expression patterns in vascular tumors including infantile hemangiomas and angiosarcomas [19–23]. Other labs have shown that β-AR signaling affects epithelial-to-mesenchymal transition [24] and β-blockade reduces tumor angiogenesis, cell proliferation, migration, and invasion [25–30]. Our labs and others have translated these retrospective and preclinical studies into the clinic to report that use of the β-blocker propranolol in patients with lethal angiosarcomas results in decreased tumor proliferation and sustained therapeutic responses [31–33].

Three β-ARs contribute to β-adrenergic signaling, and each plays distinctive and sometimes overlapping physiological roles largely based on their unique protein expression patterns in the human body [34]. Overexpression of β-ARs has been reported in oral squamous-cell and hepatocellular carcinomas, and their expression has been correlated with cervical lymph node metastasis, age, tumor size, and clinical stage [35, 36]. We hypothesized that the use of β-blockers would result in reduced proliferation rates in breast cancer. Indeed, it has previously been shown using *in vitro* models that propranolol potentiated the anti-angiogenic and anti-tumor efficacy of chemotherapy agents in breast cancer [29]. We tested this hypothesis by assessing the expression of β-AR1, 2, and 3 in breast carcinoma tissues and performing a retrospective analysis of 404 patients to compare the proliferation rates of breast tumors in patients who had taken β-blockers in the year prior to diagnosis relative to those who had not. We corroborated our retrospective findings using a prospective window of opportunity case study on a breast cancer patient and using *in vitro* cell based assays on a large panel of established breast cancer cell lines.

## RESULTS

### Β1-AR and β3-AR are overexpressed in breast cancer

**Figure 1:**
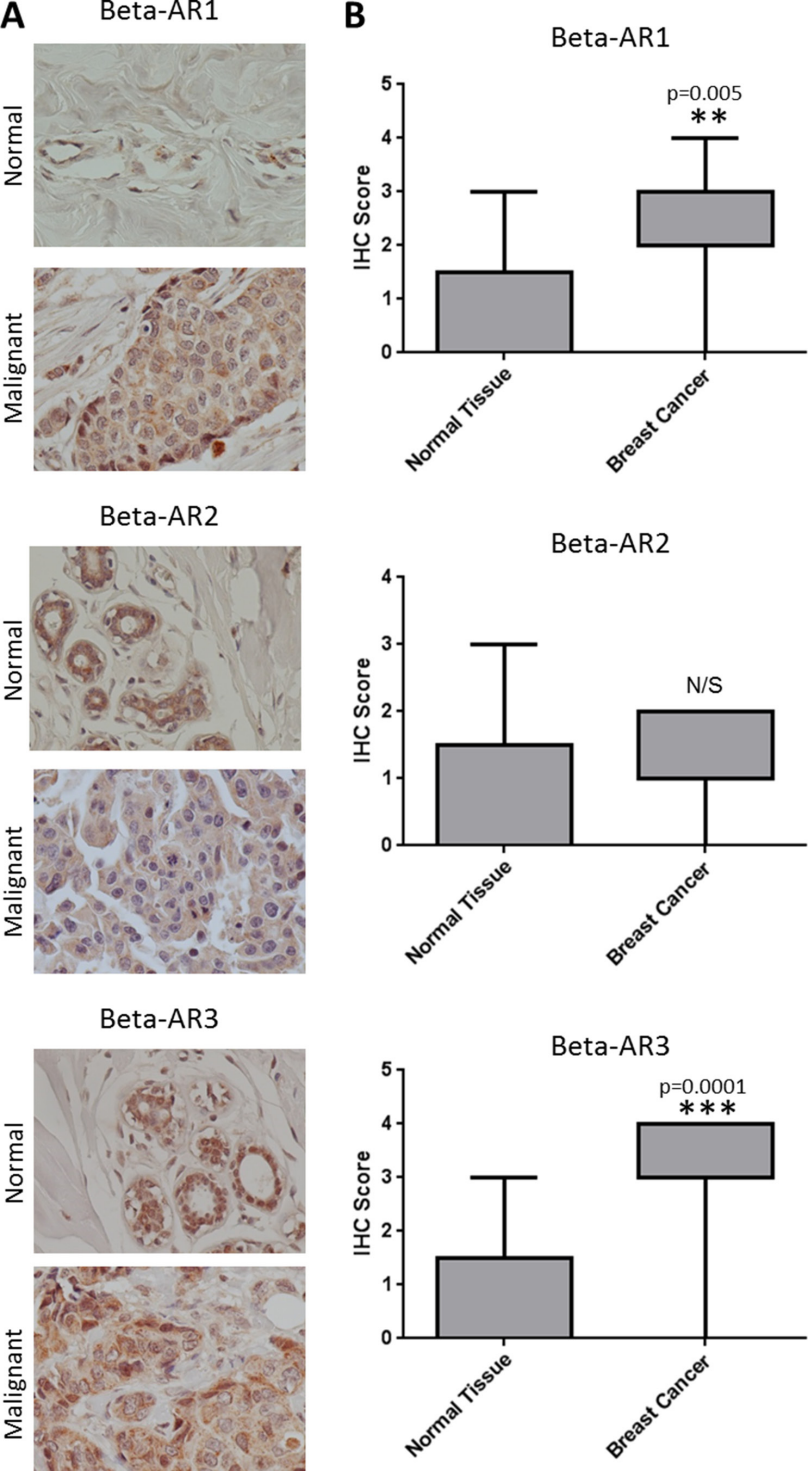
Overexpression of β-ARs in breast cancer **(A)** Representative images of IHC for the β-AR receptors in normal (N = 5) and malignant breast tissue (N = 20). **(B)** Box and whiskers plot illustrating the expression of β-ARs in the panel of normal and malignant breast tissue.

To determine if β-ARs are aberrantly expressed in breast cancer, IHC was performed on sections of normal and cancerous breast tissue. In normal breast tissue, β1-AR, β2-AR, and β3-AR staining was observed in both inner luminal epithelial cuboidal to columnar cells and outer myoepithelial contractile cells, yet largely absent in fibro-adipose tissues (Figure 1A). In breast cancer tissue, β-ARs were observed throughout the tumor cells and to a lesser degree within the tumor stroma (Figure 1A). IHC intensity scores were collected for each tissue evaluated, revealing that both β1- and β3-AR are expressed at a higher level in breast cancer relative to normal breast tissue. A difference in β2-AR expression was not detected between normal and breast cancer tissue (Figure 1B).

### Use of non-selective β-blockers is associated with reduced tumor proliferation in early stage breast cancer patients

**Figure 2:**
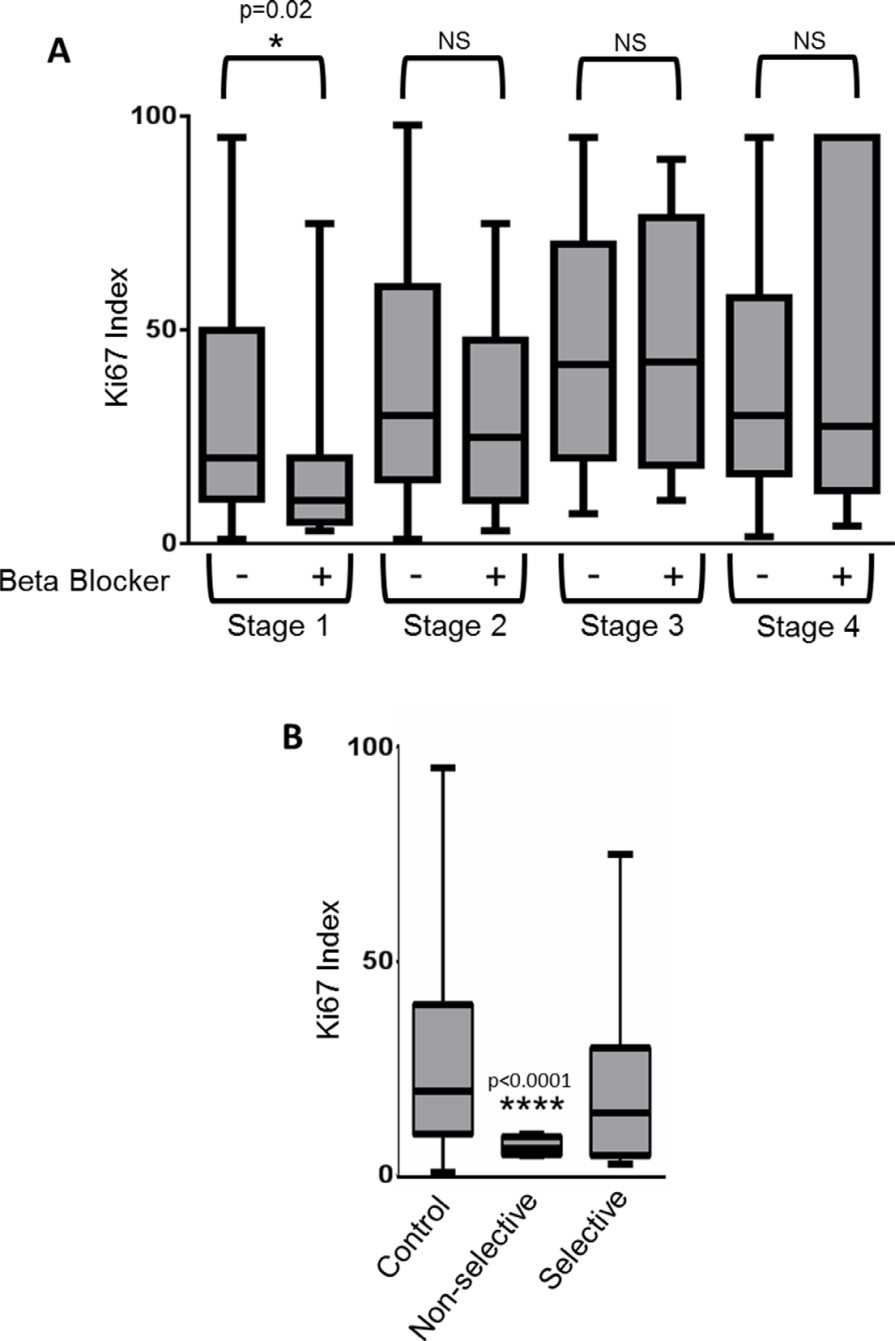
Use of β-blockers is correlated with a reduction in the proliferation rate of Stage I breast cancers **(A)** Retrospective analysis of 404 breast cancer patients was performed to determine if use of β-blockers correlated with the tumor Ki-67 index. Box and whiskers plot illustrating the lack of correlation between β-blocker use and breast tumor proliferative rate as determined by the Ki-67 index. **(B)** Stage I breast cancer patients from the retrospective study were stratified based on no β-blocker use (control), or the use of non-selective or selective β-blockers. Shown is a box and whiskers plot illustrating the significant correlation between non-selective β-blockers and reduced Ki-67 proliferative index.

We carried out a retrospective study of 404 patients diagnosed with breast cancer to assess the association between use of β-blockers and breast tumor proliferation rates. No difference was found in tumor staging or hormone receptor status between users of β-blockers and non-users (Table 1, Figure 2A). However, in patients with Stage I breast cancer, use of β-blockers revealed a significant decrease in the Ki-67 based tumor proliferative index compared to patients who were non-users of β-blockers (*p* = 0.02) (Table 1, Figure 2A). In addition, a trend towards a significant (27% decrease; *p* = 0.1096) association was observed between β-blocker usage and Ki-67 index in Stage II breast cancer.

**Table 1:** Clinicopathological features of normal and cancer breast tissues used for β-AR IHC

Characteristics	Overall	Normal	Cancer
# patient samples	25	5	20
Age [mean years (s.d.)]	48 ± 9	42 ± 7	50 ± 9
Sex	25F	5F	25F
Tumor Stage			
II	18	N/A	18
III	2	N/A	2

A recent publication suggested that only non-selective β-blockers were effective at decreasing mortality in ovarian cancer patients [15], thus we stratified our Stage I breast cancer patients based on β-AR selectivity. A significant decrease in the tumor proliferative index was observed in patients taking non-selective β-blockers (Figure 2B) (control=28.9% +/– 2.2 (SEM); non-selective β-blockers = 7.1% +/– 1.1 (SEM); *p* < 0.0001). This difference was not found in Stage II, III, or IV breast cancer patients.

### Propranolol reduced the breast cancer proliferative index in a window of opportunity case study

**Figure 3:**
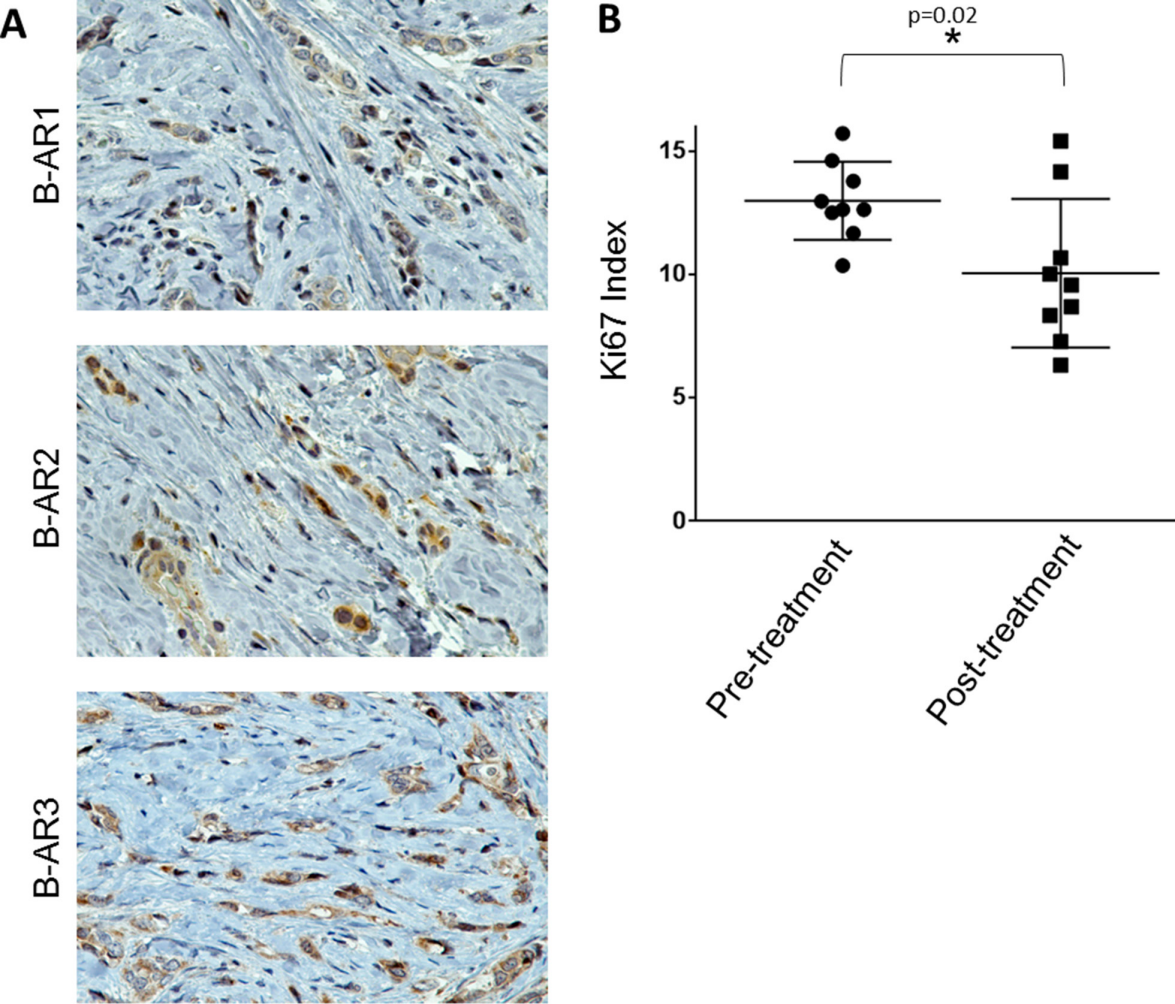
Testing the efficacy of propranolol in a prospective window of opportunity breast cancer case study **(A)** Representative 600x images of IHC for the β-AR receptors in a patient diagnosed with Stage I, hormonal receptor positive, HER2-neu negative ductal carcinoma breast cancer. **(B)** The patient was given propranolol as indicated in the materials and methods section over a 25-day period. Ki-67 IHC was performed on the diagnostic biopsy before propranolol treatment began (pre-treatment) and at the surgical resection after 25 days propranolol (post-treatment). The scatter plot depicts the Ki-67 index pre- and post-treatment across 9 vision fields with over 250 cells/field.

To prospectively test the findings from our retrospective study, we administered a non-selective β-blocker, propranolol, to a patient treated at the Texas Tech Breast Care Center. The patient had a diagnostic mammogram and ultrasound at baseline, showing a solid micro-lobulated mass with irregular margins at 1 o’clock position, measuring 1 cm in diameter. She presented with an enlarged lymph node in the left axilla (3.5 cm), and ultrasound guided biopsy of the left axillary lymph node revealed no evidence of metastatic carcinoma. Pathology revealed hormone receptor positive, HER2-neu negative invasive ductal carcinoma, 0.5 cm × 1.0 cm × 1.0 cm, moderately differentiated, with negative surgical margins. Based on immunohistochemistry, the tumor was positive for all three β-ARs (Figure 3A). Immunohistochemistry for Ki-67 was performed on the diagnostic biopsy (pre-treatment with propranolol) and on the surgical resection (post-treatment; 25 days of propranolol as indicated in the Materials and Methods section). A 23% reduction in the mean Ki-67 proliferative index was observed in the post-treatment tumor (Mean +/– SEM: 10% +/– 1.0) compared to the pre-treatment tumor (Mean +/– SEM: 13% +/– 0.5) (*p* = 0.02) (Figure 3B). Given that substantial concordance has been reported for the Ki-67 index between breast cancer core needle biopsies and their respective final surgical specimen [37], we believe this propranolol-mediated statistically significant decrease in tumor proliferation is valid.

### β-blockade reduces the proliferative index of breast cancer cell lines

**Figure 4:**
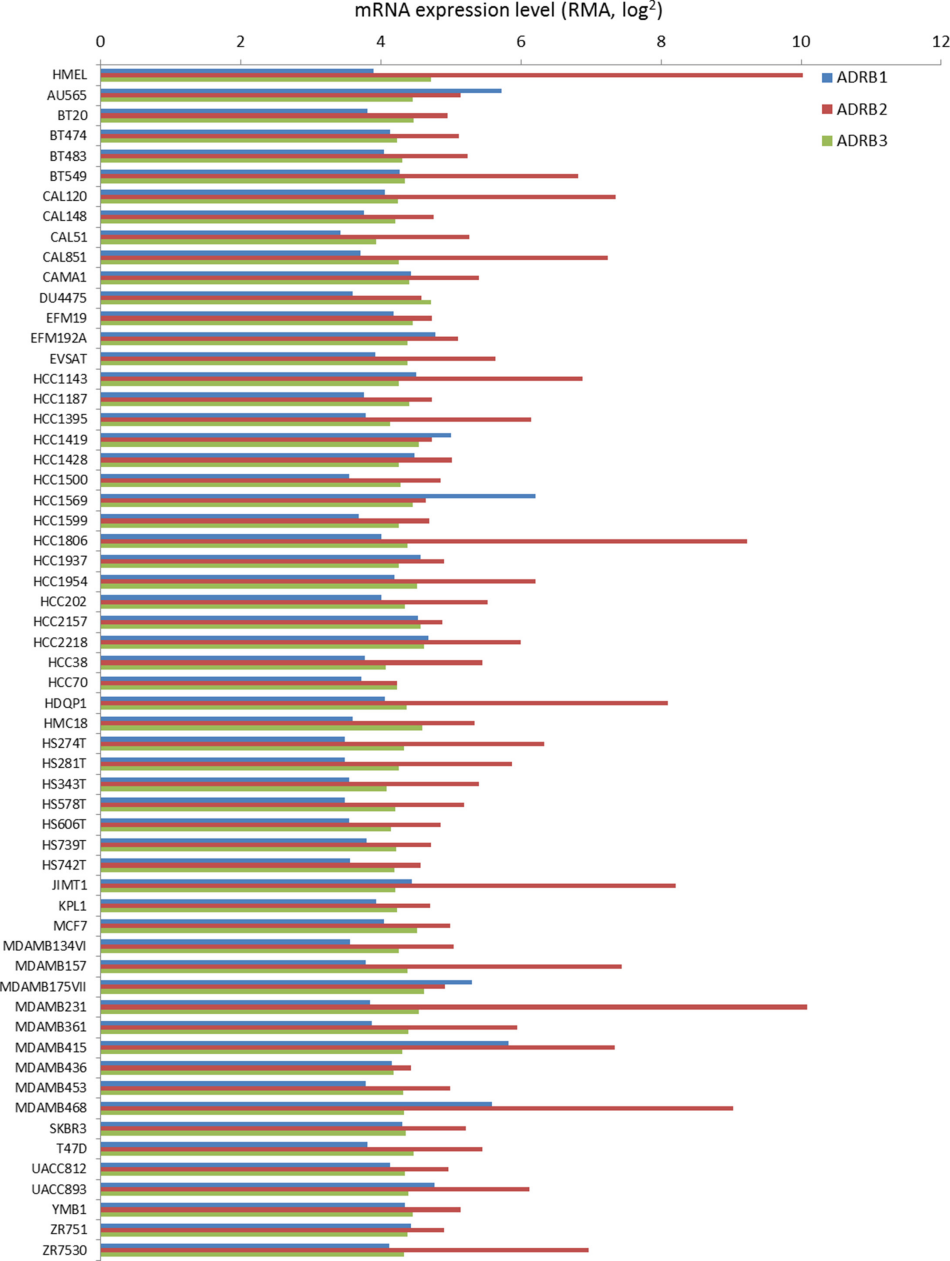
ADRB mRNA expression across a panel of normal and malignant breast cell lines mRNA expression of ADRB1, ADRB2, and ADRB3 across a panel of established breast cancer lines based on data housed in the CCLE database. Expression of the mRNAs in a human immortalized mammary epithelial cell line (HMEL) was used as a control.

**Figure 5:**
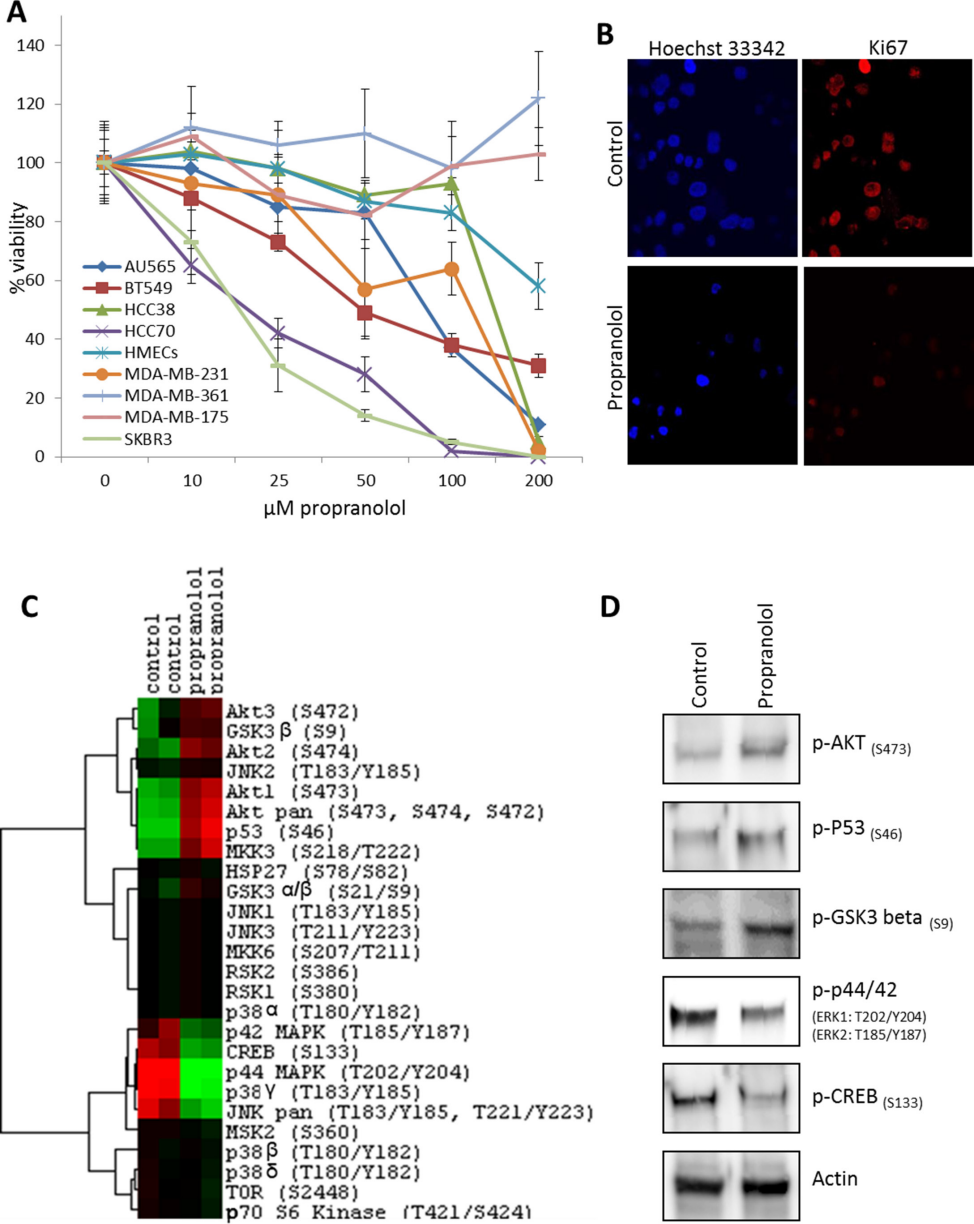
β-blockers inhibit the proliferation of breast cancer cell lines **(A)** A dose curve of propranolol was administered to a panel of breast cancer cell lines and normal primary mammary epithelial cells (HMECS) and viability was assessed. **(B)** Immunofluorescent detection of Ki-67 protein expression in control or propranolol-treated (18 μM) SK-BR-3 breast cancer cells after 24 hours. Hoechst 33342 was used as a nuclear counterstain. **(C)** Heatmap depicting phospho-MAPK antibody array results testing the status of 24 kinases in SK-BR-3 cells subjected to 24 hours control or propranolol (18 μM). (red = upregulated; green = downregulated; black = no detectable expression). **(D)** Western blot confirmation of the antibody array results in SK-BR-3 cells subjected to 24 hours control or propranolol (18 μM). (D) Western blot analysis of cell lysates from SK-BR-3 cells subjected to 24 hours control or propranolol (18 μM), confirming the phosphorylation events identified in the antibody array.

While our retrospective analysis revealed that selective β-blockers reduced the proliferative index of breast tumors, it is currently unknown whether these drugs directly affect the malignant cells within the tumor or indirectly regulate tumor proliferation through alternative mechanisms. We performed meta-analysis on a panel of established cell lines to determine the relative mRNA expression of the *ADRB*s in non-tumor versus malignant breast cancer cells. Our data revealed that *ADRB1* and *ADRB3* mRNA was consistently expressed across all cell lines tested, while *ADRB2* mRNA expression exhibited variations from one cell line to another (Figure 4). It is worth noting that *ADRB2* mRNA is the most highly expressed ADRB gene in ~90% of the cell lines tested. We subjected the panel of breast cancer cells to a dose curve of the non-selective β-blocker propranolol and measured cell viability. A primary culture of HMECs was used as a control. While cell viability of HMECs was minimally affected by even very high concentrations of propranolol (EC_50_ > 200 μM), cell viability in the panel of breast cancer cells was variably affected by the addition of propranolol with EC_50_ values ranging from 18 μM to greater than 200 μM depending on the cell line tested (Figure 5A, Table 2). Ki-67 staining of the SK-BR-3 line revealed that propranolol disrupted breast cancer cell proliferation (Figure 5B). To determine the molecular mechanism by which propranolol decreases breast cancer viability, we performed an antibody array and validation experiments to examine the phosphorylation status of a number of proteins involved in cell proliferation and survival. Our data revealed that 24 hour exposure of SK-BR-3 cells to 18 μM propranolol (this cell line's EC_50_) resulted in decreased phosphorylation of multiple mitogenic activated protein kinases (MAPKs) as well as the cAMP responsive element binding protein (CREB), and increased phosphorylation of AKT (PKB, Protein Kinase B), glycogen synthase kinase 3 (GSK3), and p53 (Figure 5C and 5D). These data suggested that propranolol induced a cellular state indicative of reduced proliferation and increased cell stress/damage.

**Table 2:** Association between β-blocker usage and clinicopathological characteristics in breast cancer patients

Characteristics	No β-Blocker *N* = 349 (86.4%)	β-Blocker *N* = 55 (13.6%)	*p* value	Significant
Tumor stage				
Stage I (# patients)	123 (30.5%)	15 (3.7%)	0.60	No
Stage II (# patients)	125 (30.9%)	19 (4.7%)		
Stage III (# patients)	76 (18.8%)	15 (3.7%)		
Stage IV (# patients)	25 (6.2%)	6 (1.5%)		
Hormonal status				
ER			0.94	No
– (# patients)	95 (23.5%)	17 (4.2%)		
+ (# patients)	249 (61.6%)	38 (9.4%)		
Unknown (# patients)	5 (1.2%)	0 (0%)		
PR			0.89	No
– (# patients)	135 (33.4%)	21 (5.2%)		
+ (# patients)	209 (51.7)	34 (8.4%)		
Unknown (# patients)	5 (1.2%)	0 (0%)		
HER2			1.00	No
– (# patients)	255 (63.1%)	41 (10.1%)		
+ (# patients)	58 (14.4%)	9 (2.2%)		
Unknown (# patients)	17 (4.2%)	5 (1.2%)		
Ki-67 index				
Stage I Mean (SEM)	28.9(2.2)	17.2(4.8)	0.02	Yes
Non-Selective β-blocker		20.9(2.2)	0.25	No
Selective β-blocker		7.1(1.1)	< 0.0001	Yes
Stage II Mean (SEM)	39.6(2.6)	31.4(4.5)	0.43	No
Stage III Mean (SEM)	44.9(3.1)	44.4(6.1)	0.95	No
Stage IV Mean (SEM)	38.3(5.3)	44.0(16.5)	0.67	No

ER = estrogen receptor.

PR = progesterone receptor.

HER2 = HER2/neu receptor.

**Figure 6:**
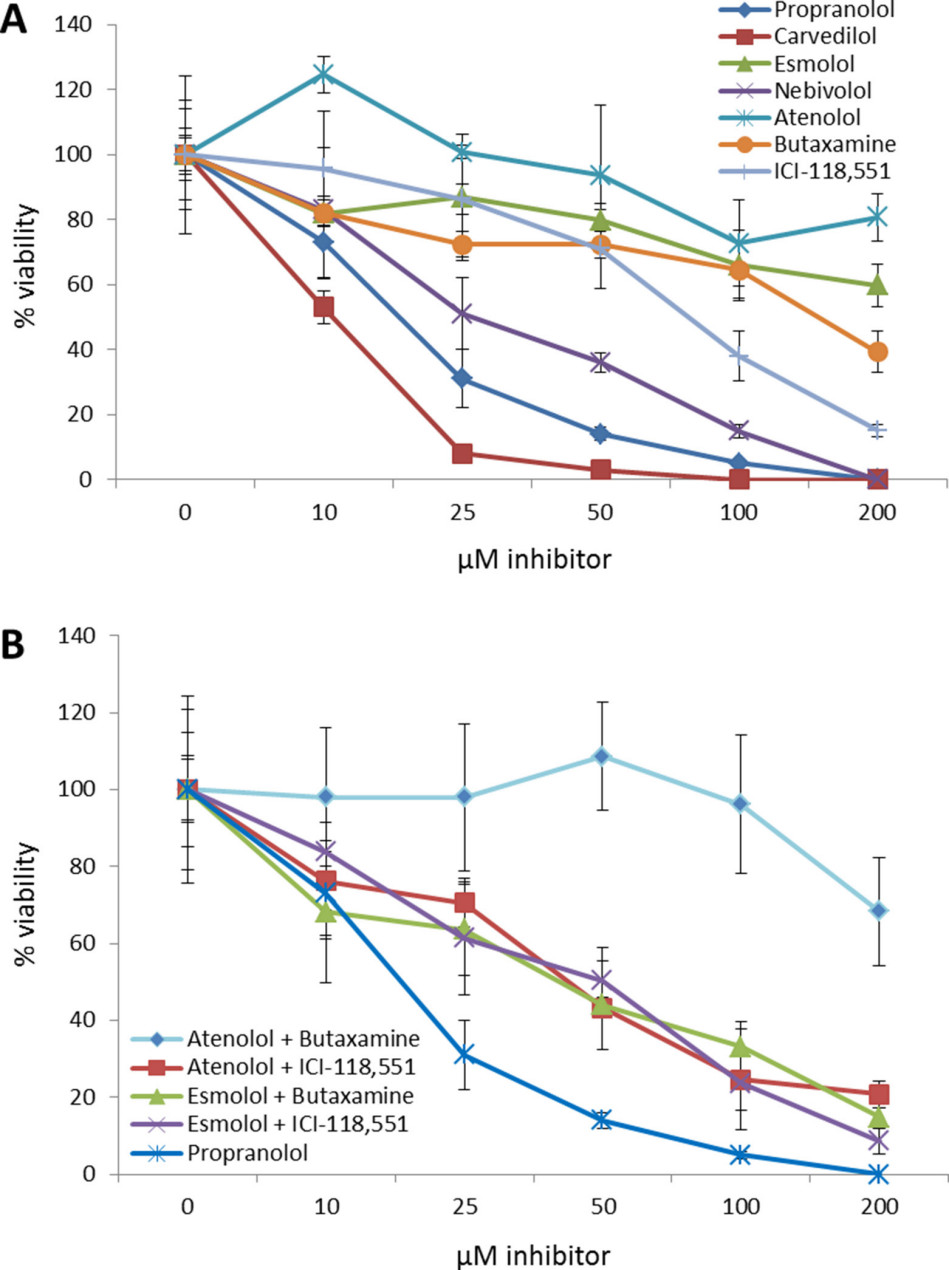
Non-selective beta blockers exhibit higher efficacy than selective beta blockers at decreasing breast cancer cell viability **(A)** Dose curves of β1-AR or β2-AR selective antagonists were administered to the SK-BR-3 breast cancer cell line and cell viability was assayed and compared to propranolol. **(B)** Dose curves of β1-AR and β2-AR selective antagonist combinations at their determined EC50 values were administered to the SK-BR-3 breast cancer cell line and cell viability was assayed and compared to propranolol.

Similar to what was observed in the retrospective patient study described above, neither β1-AR nor β2-AR selective inhibitors, when used alone, were as effective as the non-selective β-blockers propranolol or carvedilol (Figure 6A, Table 3). It should be noted that nebivolol, a β1-AR inhibitor, was more effective than any of the β-AR selective inhibitors, however this compound also exhibits a large number of off-target effects against serotonin, dopamine, histamine, and α-AR receptors. Additionally, the non-selective β-blocker carvedilol, which also is also a noted α-AR receptor inhibitor, was more effective at reducing breast cancer cell number than propranolol which targets β-AR receptors. Combinations of selective β1-AR and β2-AR inhibitors failed to produce synergy based on two independent models [38, 39] (data not shown), and these combinations, when added at their determined EC_50_ values for SK-BR-3 cells, also failed to replicate the decreases in viability observed following propranolol treatment (Figure 6B).

**Table 3:** β-blocker usage identified in the retrospective study

β-Blocker	# Patients	Dose Range	Selectivity
Carvedilol	9	2.12–25 mg daily	Non-selective
Propranolol Sotalol	1	40 mg daily	Non-selective
Timolol	1	160 mg daily	Non-selective
Acebutolol	1	0.25%, 2 drops/day	Non-selective
Atenolol	1	50 mg daily	β1-AR
Bisoprolol	12	25–100 mg daily	β1-AR
Metoprolol	1	6.25 mg daily	β1-AR
	28	25–100 mg daily	β1-AR

## DISCUSSION

In this study, we have shown that all three β-ARs are expressed in breast cancer tissue, and β1-AR and β3-AR are overexpressed in breast cancer relative to normal breast tissue. In addition, this study suggests that use of non-selective β-blockers in patients with early stage breast cancer may lead to decreased tumor proliferation.

Aberrant expression of β2-AR is associated with promoting the oncogenic properties of breast cancer, increasing axillary lymph node metastasis, and leading to poor disease free survival [40, 41]. β2-AR potentially mediates these processes through activating a cAMP-calcium feed-forward loop to drive breast cancer cell invasion and promote invadopodia formation to enhance breast cancer cell invasion [42, 43]. While β2-AR undoubtedly plays an important role in breast cancer, our data show that β1-AR and β3-AR are expressed more in breast cancer tissue relative to normal mammary epithelium at the protein level. Indeed, β1-AR and β3-AR are reportedly involved in cancer processes, whereby β1-AR has been shown to contribute to enhanced lipolysis in cancer cachexia [44] and β3-AR missense mutations have been correlated with obesity related breast cancer in African-Americans [45]. ADRB mRNA expression was not significantly different between normal and cancer cell lines, suggesting some form of translational or post-translational regulation likely contributes to the differential expression of β1-AR and β3-AR protein levels between the normal and malignant tissues.

A wealth of retrospective reports supports the therapeutic utility of β-blockers in breast cancer and other malignancies [6–15]. While many of these retrospective studies examined therapeutic endpoints such as progression free survival and patient mortality, none have mechanistically correlated tumor response at the molecular level to β-blockade. Preclinical studies in diverse cancers have revealed that β-blockade decreases tumor cell proliferation rates [21, 27, 46–49]. In this study we sought to determine if β-blockade affects tumor cell proliferation in a large patient population stricken with a very common tumor type. Our data showed significant association between the use of non-selective β-blockers and breast cancer proliferation rate, revealing an over 75% reduction in the proliferation rate of Stage I breast cancer following use of non-selective β-blockers such as carvedilol, propranolol, or sotalol. These data suggest a possible use for non-selective β-blockers in breast cancer prevention or in the treatment of early stage disease before the development of advanced tumors. Moreover, the efficacy of non-selective over selective β-blockers corroborates previously reported data revealing that non-selective β-blockers, but not selective β-blockers, were effective at reducing mortality in ovarian cancer patients [15].

Our *in vitro* cell line-based data demonstrated that there was no correlation between the sensitivity of the breast cancer cell lines for beta blockade and their respective levels of β-AR mRNA expression. In cell lines we corroborated our retrospective and prospective analyses, showing that non-selective beta blockade was more effective at reducing breast cancer cell viability than selective beta blockade. Based on our expression data, both of the non-selective beta blockers, propranolol and carvedilol, were more effective than any selective beta blocker, and we suspect the increased efficacy of carvedilol over propranolol was based on carvedilol's added alpha-1 adrenergic (α1-AR) receptor inhibitor activity (K_i_ = 2.2 nM). Only nebivolol, a third generation β1-AR selective inhibitor, came close to recapitulating the activity of the non-selective beta blockers, however this compound exhibits the highest β1-AR affinity among β-blockers, uniquely stimulates the activity of nitric oxide synthase, and has a number of off-target effects including α1-AR (K_i_ = 1.2 μM), serotonin 5-HT1a (K_i_ = 20 nM), serotonin 5-HT2 (K_i_=700 nM), dopamine (K_i_ = 4 μM), and histamine H1 receptor (K_i_ = 2.4 μM) which may contribute to its enhanced efficacy relative to other selective β1-AR inhibitors. Indeed, many of these off-target pathways are reportedly novel anti-cancer targets [50–53]. No synergism was calculated based on combinations of β1-AR and β2-AR antagonists, and these combinations were incapable of recapitulating the anti-proliferative effects of the non-selective β-blocker propranolol, suggesting the potential that off target effects partially contribute to the effectiveness of non-selective beta blockers against breast cancer cell lines. As mentioned above, carvedilol exhibits off target effects on β1-AR, and propranolol demonstrates high affinity for serotonin 5-HT1B receptors (K_i_ = 17 nM) and mild affinity for serotonin 5HT-1D receptors (K_i_=10.2 μM). In summary, future prospective clinical trials testing β-blockers in breast cancer should focus on using non-selective β-blockers such as propranolol or non-selective α-AR/β-AR inhibitors such as carvedilol against early stage disease.

Our retrospective data analysis has some limitations. First, due to retrospective data collection, we could not determine the long-term effect of using β-blockers. Second, our data comprised only a small proportion of β-blocker users (~13% of the patients). Therefore, we could not adjust for any differences in the baseline cofactors while determining association of β-blockers with Ki-67. Third, we could not collect any other medication data or commodities data which might modify the effect of β-blockers on Ki-67. Nonetheless, our retrospective data analysis along with laboratory experimental studies, prospective study, and bioinformatics meta-analysis study provide ample evidence that non-selective β-blockers could be effective in reducing Ki-67 among early stage breast cancer. Moreover, interpretation of a single prospective case report must be taken with caution and further clinical studies are needed to corroborate these findings.

If the wealth of preclinical, retrospective, and clinical data continue to support the use of β-blockers as a supplement to existing anti-cancer therapeutic regimes, this class of drugs could be used as safe, low cost therapeutics that are especially useful in the era of prohibitively costly new anti-cancer therapeutics, most particularly in low resource settings where many cancer patients do not possess the financial means or access to expensive novel treatments.

## MATERIALS AND METHODS

### Immunohistochemical analysis of β-AR expression in breast tissue

Immunohistochemistry (IHC) for β1-AR, β2-AR, and β3-AR expression was performed on tissue arrays composed of five normal, fibrofatty breast tissues and twenty invasive, ductal carcinoma tissues (US Biomax, Cat# MC5003b). The known clinicopathological features associated with these tissues are described in Table 4. Sections were deparaffinized, rehydrated, and treated for antigen retrieval using Trilogy (Cell Marque). Nonspecific binding was blocked with background block solution (Cell Marque). The following primary antibodies were used for antigen detection: anti-β1-AR (1:200, Abbiotech Cat# 250919), anti-β2-AR (1:200, Abbiotech Cat# 251604), anti-β3-AR (1:200, Abbiotech Cat# 251604), and anti-Ki-67 (1:200, Abcam Cat# ab15580). Each anti-β-AR antibody was raised against synthetic peptides unique and non-overlapping for each protein. Specificity was tested based on western blotting bands from cancer cell line lysates matching to the appropriate molecular weight for each protein; no overlap in antigenicity was observed (data not shown). Sections were incubated with the CytoScan Alkaline Phos Detection System (Cell Marque) and detected using the DAB substrate kit (Cell Marque). All slides were counterstained with hematoxylin. Immunopositive staining was semi-quantitatively evaluated by 2 individuals blinded to sample identification based on staining intensity within the tumor-positive areas of the tissue.

**Table 4:** EC_50_ values for propranolol in normal and malignant breast cell lines

Cell Line	EC_50_
HMEC	> 200 μM
AU565	81 μM
BT549	50 μM
HCC38	93 μM
HCC70	20 μM
MDA-MB-175	> 200 μM
MDA-MB-231	78 μM
MDA-MB-361	> 200 μM
SK-BR-3	18 μM

### Retrospective study

Analysis of 404 female patients diagnosed with invasive ductal carcinoma at the Texas Tech Breast Care Center between the years of 2005 to 2014 was performed. 90.1% of the patients were Hispanic. IHC for estrogen receptor (ER), progesterone receptor (PR), Her-2/neu, and Ki-67 tumor proliferative index was available for all patients. Breast cancer clinico-pathological features (age at diagnosis, tumor size, tumor grade, lymph node status, hormonal receptor status) were extracted from each case report, and these data are presented in Table 1. Patients were considered positive for β blocker usage if they had been prescribed β-blockers at any point in the year immediately prior to diagnosis. In total, we identified 54 patients taking β-blockers (61.0 +/– 10 years old) and 350 patients (54.6 +/– 12 years old) that were not taking β-blockers during the defined time frame. Non-selective β-blockers were prescribed to 22% of the patients, while 78% of the patients were prescribed selective β-blockers (Table 5).

**Table 5:** EC_50_ values for selective and non-selective β-blockers in SK-BR-3 breast cancer cells

β-blocker	Selectivity	EC_50_
Propranolol	Non-selective	18 μM
Carvedilol	Non-Selective	11 μM
Esmolol	β1-AR	> 200 μM
Nebivolol	β1-AR	32 μM
Atenolol	β1-AR	> 200 μM
Butaxamine	β2-AR	144 μM
ICI-118,551	β2-AR	81 μM

### Prospective window of opportunity case study

A patient diagnosed with Stage I, hormonal receptor positive, HER2-neu negative ductal carcinoma was seen and enrolled in the study at the Texas Tech Breast Care Center. The patient was prescribed 1.5 mg/kg/day propranolol for 18 days and then a tapering dose over the subsequent 7 days. The tapering dose was required by IRB and after consultation with a cardiologist. In total, the patient was administered propranolol for 25 days. The dose of propranolol used in this study was within the typical range of 80–320 mg per day prescribed for ailments such as hypertension, angina pectoris, myocardial infarction, and hemangioma. IHC was performed to confirm the expression of all three β-ARs. Ki-67 immunohistochemistry (1:200, Abcam Cat# ab15580) was performed on tissue sections from the diagnostic biopsy (pre-treatment with propranolol) and on the surgical resection of the tumor (post-treatment with propranolol). The Ki-67 index was blindly quantified in both the pre- and post-treatment tissues by counting 9 fields under a microscope, with each field composed of 250+ nuclei. The Ki-67 index was calculated as the (number of Ki-67 positive nuclei/total number of nuclei) × 100.

### Meta-analysis of breast cancer cell line genomics data

Quantile normalized mRNA expression (Affymetrix U133+2 arrays) for human breast cancer and immortalized mammary epithelial (HMEL) cell lines were downloaded from the Broad Institute Cancer Cell Line Encyclopedia (CCLE) database ( http://www.broadinstitute.org/ccle) [54]. The data were processed using GENE-E (The Broad Institute of MIT and Harvard, http://www.broadinstitute.org/cancer/software/GENE-E).

### Viability assays

Normal breast and breast cancer cell lines were cultured in incubators maintained at 37 degrees Celsius in the presence of 5% CO_2_. Primary cultures of normal human mammary epithelial cells (HMECs; ATCC #PCS-600–010) were grown in the EBM-2 bullet kit (Lonza) with penicillin/streptomycin antibiotics. MDA-MB-361 (ATCC #HTB-27) was grown in Leibovitz's L-15 medium supplemented with 20% fetal bovine serum and penicillin/streptomycin antibiotics. AU565 (ATCC #CRL-2351), HCC70 (ATCC #CRL-2115), BT-549 (ATCC #HTB-122), HCC38 (ATCC # CRL-2314), and MDA-MB-231 (ATCC #HTB-26) were grown in RPMI 1640 medium supplemented with 10% fetal bovine serum and penicillin/streptomycin antibiotics. SK-BR-3 (ATCC #HTB-30) was grown in McCoy's medium supplemented with 10% fetal bovine serum and penicillin/streptomycin antibiotics. All cell lines were used for less than 6 months from the date of purchase and were characterized by ATCC based on morphology, karyotyping, and PCR based approaches to confirm their identify and rule out intra- and inter-species contamination. DL-propranolol hydrochloride (Acros Organics), carvedilol (Tocris Bioscience), esmolol (Santa Cruz Biotechnology), nebivolol (Santa Cruz Biotechnology), atenolol (Acros Organics), butaxamine hydrochloride (Santa Cruz Biotechnology), and ICI 118,551 hydrochloride (Santa Cruz Biotechnology) was added to the cell lines at the indicated concentrations, and cell viability was assessed after 48 hours by alamar blue cell viability assays (ThermoScientific). Two models, the Bliss Independence Model [38] and the Linear Interaction Effect Model [39], were used to evaluate drug combination synergy.

### Immunofluorescence

SK-BR-3 cells were treated with propranolol for 24 hours and subsequently fixed in 4% paraformaldehyde. Cells were permeabilized with Triton X-100 and incubated with an anti-Ki-67 antibody (1:400, Abcam Cat# ab15580) and anti-rabbit secondary antibodies conjugated to AlexaFlour 594 dye (ThermoFisher). Hoechst 33342 was used as a nuclear stain. Confocal images were obtained using a Nikon Eclipse T*i*.

### Antibody array

The Phospho-Mitogen-activated Protein Kinase (MAPK) Antibody Array (R&D Systems #ARY002B) was performed on SK-BR-3 cells treated for 24 hours with either a control or 18 μM propranolol according to the manufacturer's instructions. Data was centered, normalized, and clustered using a centroid linkage via an un-centered similarity metric with Cluster 3.0 software. Heatmaps were visualized using Java Treeview software.

### Western blotting

Protein extracts from SK-BR-3 cells were separated by SDS-PAGE, transferred onto PVDF membranes, and probed with antibodies against phospho-AKT (S473) (Cell Signaling #9271), phospho-p53 (S46) (Cell Signaling #2521), phospho-GSK3β (S9) (Cell Signaling #5558), phospho-p44/42 (ERK1: T202/Y204; ERK2: T185/Y187) (R&D Systems #AF1018), phospho-CREB (S133) (Cell Signaling #9196), and actin (Santa Cruz Biotechnology #sc47778). Proteins were detected with HRP-conjugated secondary antibodies and visualized with the Pierce ECL Western blotting substrate (ThermoScientific) according to the manufacturer's protocol.

### Statistical analysis

For statistical analysis of β-AR expression in breast tissue, the Mann-Whitney rank sum test was used. For the retrospective study of breast cancer patient data, the relationship between β-blocker usage and the Ki-67-based proliferative index of the breast tumors was determined with the Mann-Whitney rank sum test. Ki-67 was used to measure tumor proliferation given its established role as a proliferative marker [55], and we have previously demonstrated that the expression of this protein provides a very good concordance with the Recurrence Score Pathology-Clinical (RSPC value) in breast cancer in our pathology lab [56]. Unpaired *t-test* was used to compare the age distribution between β-blocker users and non-users. Comparisons of β-blocker usage to tumor hormonal receptor status and tumor staging were calculated with the Fisher's exact test. Paired *t-test*s were used to compare Ki- 67 expression in the prospective pre- and post-treatment breast cancer patient sample. Statistical analyses were carried out using STATA 13. Differences were considered statistically significant if the *p value* was less than 0.05.
